# Identifying the relationship between hospital rurality and antibiotic overuse

**DOI:** 10.1017/ash.2023.263

**Published:** 2023-09-29

**Authors:** Hannah Hardin, Valerie Vaughn, Andrea White, Jennifer Horowitz, Elizabeth McLaughlin, Julia Szymczak, Lindsay Petty, Anurag Malani, Scott Flanders, Tejal Gandhi

## Abstract

**Background:** Antibiotic overuse and the resulting patient outcomes span all hospitals. However, although antibiotic stewardship can improve antibiotic use, effective stewardship programs require expertise and an infrastructure that are not present in all hospitals. Rural hospitals have less access to resources, infectious disease expertise, and participation in academic research. Thus, we compared antibiotic overuse at discharge between rural and nonrural hospitals for patients diagnosed with community-associated pneumonia (CAP) or urinary tract infection (UTI)—the 2 most common hospital infections. **Methods:** To determine whether antibiotic overuse at discharge was higher among rural versus nonrural hospitals, we analyzed data from a 41-hospital prospective cohort of patients treated for CAP or UTI between July 1, 2017, and July 30, 2019, in Michigan. Antibiotic overuse was defined as treatment that was unnecessary (ie, patient did not have an infection), excessive (ie, duration >4 days for CAP), or included suboptimal fluoroquinolone use (ie, safer alternative available). Overuse was determined based on patient risk factors, symptoms, allergies, diagnostic results, and time to stability. Hospital rurality was defined using the Rural–Urban Continuum Codes (RUCC) score. We defined rural as a score ≥4 and very rural as a score of 7–9. We used *t* tests to compare the mean percentage of patients with antibiotic overuse at discharge between nonrural and rural (and very rural) hospitals. **Results:** Across 41 hospitals, we included 23,449 patients with CAP or UTI. There were 5 rural (and 3 very rural) hospitals with 2,039 (and 1,082) patients. Antibiotic overuse at discharge was present in 43.1% of patient cases in nonrural hospitals, 52.5% in rural hospitals (*P* = .04 vs nonrural) and 58.1% in very rural hospitals (*P* = .007 vs nonrural). Compared to nonrural hospitals, the mean percentage of cases with antibiotic overuse at discharge in rural hospitals was 9.4% higher (15.1% higher in very rural hospitals). Results were similar in a subgroup analysis of only patients with UTI (47.0% in rural vs 37.5% in nonrural, mean difference, 9.5%; *P* = .03) but were not statistically significant in patients with CAP (53.8% vs 48.0%, respectively; mean difference, 5.8%; *P* = 0.23). **Conclusions:** In this retrospective study, rural hospitals—especially very rural hospitals, had higher rates of antibiotic overuse at discharge than nonrural hospitals. Our findings suggest that antibiotic stewardship interventions tailored toward the unique differences in infrastructure, resources, and needs of rural hospitals are essential to community health.

**Figure:**
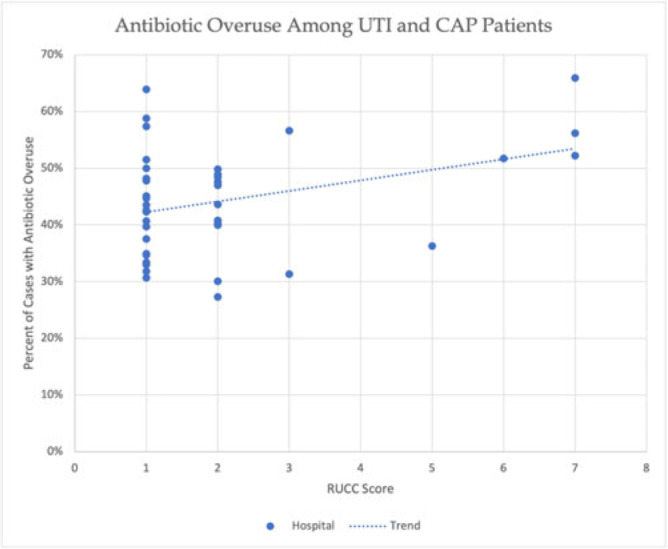

**Disclosures:** None

